# Association between GWAS-Identified Genetic Variations and Disease Prognosis for Patients with Colorectal Cancer

**DOI:** 10.1371/journal.pone.0119649

**Published:** 2015-03-23

**Authors:** Byung Woog Kang, Hyo-Sung Jeon, Yee Soo Chae, Soo Jung Lee, Jae Yong Park, Jin Eun Choi, Jun Seok Park, Gyu Seog Choi, Jong Gwang Kim

**Affiliations:** 1 Department of Oncology/Hematology, Kyungpook National University Medical Center, Kyungpook National University School of Medicine, Daegu, Republic of Korea; 2 Department of Molecular diagnostics and Imaging research institute, Kyungpook National University School of Medicine, Daegu, Republic of Korea; 3 Department of Biochemistry and Cell biology, Kyungpook National University School of Medicine, Daegu, Republic of Korea; 4 Department of Internal Medicine, Kyungpook National University School of Medicine, Daegu, Republic of Korea; 5 Colorectal Cancer Center, Kyungpook National University Medical Center, Kyungpook National University School of Medicine, Daegu, Republic of Korea; Singapore General Hospital, SINGAPORE

## Abstract

Genome-wide association studies (GWASs) have already identified at least 22 common susceptibility loci associated with an increased risk of colorectal cancer (CRC). This study examined the relationship between these single nucleotide polymorphisms (SNPs) and the clinical outcomes of patients with colorectal cancer. Seven hundred seventy-six patients with surgically resected colorectal adenocarcinoma were enrolled in the present study. Twenty-two of the GWAS-identified SNPs were genotyped using a Sequenom MassARRAY. Among the 22 SNPs, two (rs1321311G>T in *CDKN1A* and rs10411210C>T in *RHPN2*) were significantly associated with the survival outcomes of CRC in a multivariate survival analysis. In a recessive model, the rs1321311 TT genotype (vs. GG + GT) and rs10411210 TT genotype (vs. CC + CT) were associated with a worse prognosis for disease-free survival (adjusted HR = 1.90; 95% confidence interval = 1.00-3.60; *P* = 0.050, adjusted HR = 1.94; 95% confidence interval = 1.05-3.57; *P* = 0.034, respectively) and overall survival (adjusted HR = 2.05; 95% confidence interval = 1.00-4.20; *P* = 0.049, adjusted HR = 2.06; 95% confidence interval = 1.05-4.05; *P* = 0.036, respectively). None of the other SNPs was significantly associated with any clinicopathologic features or survival. The present results suggest that the genetic variants of the *CDKN1A* (rs1321311) and *RHPN2* (rs10411210) genes can be used as prognostic biomarkers for patients with surgically resected colorectal cancer.

## Introduction

Although the benefit of adjuvant chemotherapy in resected colorectal cancer (CRC) has already been established by several studies, 40% of patients experience local or distant recurrences even after curative resection [1]. In particular, the heterogeneity of CRC is considered one of major difficulties for choosing treatments and the effect of chemotherapy seems to vary depending on the tumor biology. Thus, a clinical or biologic biomarker that predicts recurrence, survival, or response patterns to chemotherapy is needed for patients with resected CRC.

Single nucleotide polymorphisms (SNPs) have already been widely implicated in cancer development, prognosis, and treatment response. Recent advances using genome-wide association studies (GWASs) have enabled the identification of multiple CRC-related SNPs [2–8]. Notwithstanding, various studies have also reported that genetic variants generated from candidate gene or pathway-based studies are associated with the clinical outcome of CRC patients, which can be used to categorize patients with different survival or responses to specific treatments. However, no GWAS has yet examined the direct relationship between genetic variations and the survival of CRC patients. Three studies have examined the relationship between GWAS-identified CRC risk variants and the clinical outcomes of CRC, yet two were performed with a small sample size and the third was focused on Caucasian populations, which differ from other ethnic groups [9–11]. Accordingly, the present study examined the relationship between GWAS-identified genetic variants and the clinical outcomes of a relatively large group of Korean CRC patients.

## Materials and Methods

### Study population

The tissues investigated in this study were obtained from 776 Korean patients who underwent a curative surgical resection between May, 2001 and May, 2008 at Kyungpook National University Hospital (Daegu, Korea). Medical records were retrospectively reviewed to identify the relationship between GWAS-identified genetic variants and the clinical outcomes. In brief, the study included patients with histologically confirmed adenocarcinoma of the colon and rectum who underwent curative surgery but did not receive neoadjuvant therapy before surgery. Each patient was examined every 3 to 6 months for the first three years following the diagnosis of CRC, and every year thereafter, in accordance with the national guidelines. Written informed consent for genetic analysis including gene expression and SNP genotyping was received from all the patients before surgery, and the study was approved by the Institutional Research Board at Kyungpook National University Hospital. The CRC diagnosis and staging were assessed according to the WHO classifications and TMN classifications from the 7th edition of the American Joint Committee on Cancer (AJCC).

### SNP selection and genotyping

Thirty-five polymorphisms associated with CRC susceptibility were selected from the US National Human Genome Research Institute (NHGRI) Catalog of Published Genome-Wide Association Studies (as accessed in March 2013). Among these 35 polymorphisms, 22 polymorphisms were applied in this study, while the others were excluded for the following reasons: the minor allele frequency was ≤ 5% in the HapMap JPT data of the public SNP database (http://www.ncbi.nlm.nih.gov/SNP) or the SNPs could not be applied to the SEQUENOM’s MassARRAY platform. All the obtained tissues were immediately snapped frozen in liquid nitrogen and stored -80°C until the relevant experiments were conducted. The genomic DNA was extracted from fresh colorectal mucosal tissue at the time of surgery using a QIAamp genomic DNA kit (Qiagen, Hilden, Germany) according to the manufacturer’s protocol. The genotype analysis was performed using SEQUENOM’s MassARRAY iPLEX assay according to the instructions of the manufacturer. To validate the genotyping, approximately 5% of samples were randomly selected for re-genotyping using a sequencing method or restriction fragment length polymorphism (RFLP) assay by a different investigator and the results were 100% concordant.

### Statistical analysis

The Hardy-Weinberg equilibrium was tested using a goodness-of-fit χ2 test with 1 df. The genotypes for each SNP were analyzed as a three-group categorical variable (referent model), and also grouped according to a dominant and recessive model. Overall survival (OS) was measured from the day of surgery to the date of the last follow-up or date of death. Disease-free survival (DFS) was calculated from the day of surgery until recurrence or death from any cause. The survival estimates were calculated using the Kaplan-Meier method. The differences in OS or DFS according to the SNPs were compared using log-rank tests. Cox’s proportional hazard regression model was used for the multivariate survival analyses, which were always adjusted for age (< 64 versus ≥ 64 years), the preoperative CEA level (normal versus elevated), differentiation (well and moderate versus poorly), sex (male versus female), primary site (colon versus rectum), and stage (I to III). The proportional hazards assumption was checked and all graphs for the log-log survivor functions of the two SNP aid groups (rs10411210 and rs1321311) were parallel except for DFS graph of rs10411210. The hazard ratio (HR) and 95% confidence interval (CI) were also estimated. A cut-off *P* value of 0.05 (two-sided) was adopted for all the statistical analyses. The statistical data were obtained using an SPSS software package (SPSS 11.5 Inc. Chicago, IL, USA) or SAS Genetic software (SAS Institute, Cary, NC).

## Results

### Patient and disease characteristics

The clinical and pathologic characteristics of the patients are listed in Table 1. The median age was 64 years (range 21–89), 444 (57.2%) patients were diagnosed with colon cancer, and 327 (42.1%) patients had rectal cancer. The pathologic stages after surgical resection were as follows: stage I (*N* = 141, 18.2%), stage II (*N* = 318, 41.0%), and stage III (*N* = 317, 40.9%). Among the 635 patients with stage II or III diseases, 570 patients (89.8%) received adjuvant chemotherapy based on 6 cycles of 5-fluorouracil/leucovorin ± radiotherapy (390), 12 cycles of 5-fluorouracil/leucovorin/oxaliplatin (n = 67), 8 cycles of capecitabine (n = 41), or doxifluridine for 1 year (n = 72).

**Table 1:** Patient characteristics (*N* = 776).

Variables	*N*	%
Age, median (years)	64	
<64	378	48.7
≥64	398	51.3
Sex		
Male	432	55.7
Female	344	44.3
Primary site		
Colon	444	57.2
Rectum	327	42.1
Colon+ Rectum	5	0.6
Histological differentiation		
Well	94	12.1
Moderate	656	84.6
Poor or signet ring	26	3.4
CEA		
Normal	642	82.7
Elevated	134	17.3
CA19–9		
Normal	675	87.0
Elevated	101	13.0
Pathologic stage^a^		
I	141	18.2
II	318	41.0
III	317	40.9
Relapse		
No	545	70.2
Yes	231	29.8
Death		
No	593	76.4
Yes	183	23.6

^a^ the 7^th^ AJCC staging system

### Overall treatment outcomes

At the last overall analysis (December 2012), 231 patients had experienced a disease relapse, 163 patients had died as a result of CRC, and 20 patients had died from causes unrelated to CRC. At the median follow-up duration of 78.5 months, the estimated 5-year OS and DFS for all the patients was 83.1% and 76.0% respectively, plus the survival differed according to the stage (P<0.001).

### Genotype frequencies and effects on survival

**Fig 1:**
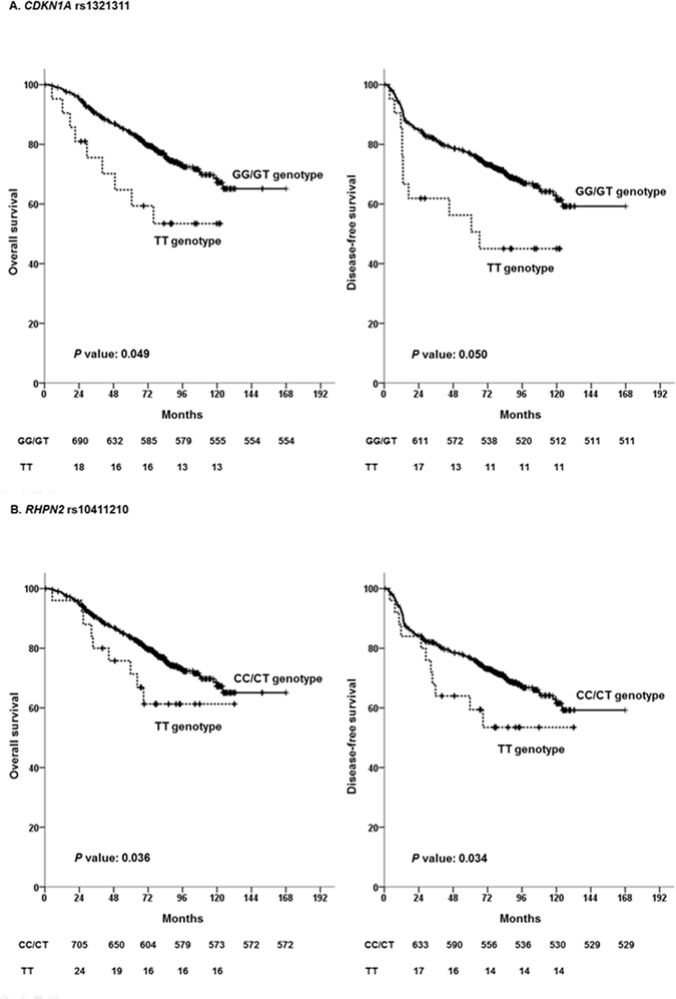
Survival curves according to genotype in combined analysis: *CDKN1A* rs1321311 (A) and *RHPN2* rs10411210 (B). *P* values correspond to multivariate Cox model adjusted for age, CEA level, differentiation, sex, primary site, and pathologic stage.

The frequencies of each genotype are shown in Table 2 and were confirmed to be in Hardy-Weinberg equilibrium (*P*>0.05). Among the 22 SNPs (S1 Table and S2 Table), two SNPs (rs1321311G>T in Cyclin-dependent kinase inhibitor 1A (*CDKN1A*) and rs10411210C>T in Rho GTPase binding protein 2 (*RHPN2*) were found to be significantly associated with the survival outcomes of CRC in the multivariate survival analysis. In the recessive model, the rs1321311 TT genotype (vs. GG + GT) and rs10411210 TT genotype (vs. CC + CT) were associated with a worse prognosis for DFS (adjusted HR = 1.90; 95% confidence interval = 1.00–3.60; *P* = 0.050, adjusted HR = 1.94; 95% confidence interval = 1.05–3.57; *P* = 0.034, respectively) and OS (adjusted HR = 2.05; 95% confidence interval = 1.00–4.20; *P* = 0.049, adjusted HR = 2.06; 95% confidence interval = 1.05–4.05; *P* = 0.036, respectively) (Table 3 and Fig. 1). None of the other SNPs was significantly associated with survival. With regard to clinicopathologic parameters, age (adjusted HR for DFS = 1.77; *P*<0.001, adjusted HR for OS = 2.56; *P*<0.001), preoperative CEA level (adjusted HR for DFS = 1.81; *P*<0.001, adjusted HR for OS = 1.78; *P* = 0.002), and pathologic stage (adjusted HR for DFS = 2.75; *P*<0.001, adjusted HR for OS = 2.49; P<0.001) were identified as significantly associated with DFS and OS (Table 3).

**Table 2:** Information and genotypes of 22 polymorphisms.

rsID	Base change	Chromosome	Gene	Position	1	2	3	MAF	*HWE-P*
rs10411210	C/T	19	RHPN2	19q13.11	527 (68.8)	214 (27.9)	25 (3.3)	0.172	0.568
rs10795668	G/A	10	KRT8P16—TCEB1P3	10p14	348 (45.1)	336 (43.5)	88 (11.4)	0.332	0.614
rs10936599	T/C	3	MYNN	3q26.2	266 (34.3)	372 (48)	137 (17.7)	0.417	0.725
rs11169552	C/T	12	DIP2B, ATF1	12q13.12	334 (44.7)	334 (44.7)	79 (10.6)	0.329	0.739
rs1321311	G/T	6	SRSF3—CDKN1A	6p21.2	534 (71.7)	189 (25.4)	22 (3)	0.156	0.294
rs3802842	A/C	11	C11orf93	11q23.1	237 (30.7)	397 (51.4)	139 (18)	0.437	0.222
rs3824999	A/C	11	POLD3	11q13.4	284 (36.8)	365 (47.3)	122 (15.8)	0.395	0.793
rs4444235	C/T	14	RPS3AP46—BMP4	14q22.2	249 (32.2)	367 (47.5)	157 (20.3)	0.440	0.306
rs4779584	T/C	15	SCG5—GREM1	15q13.3	551 (73)	182 (24.1)	22 (2.9)	0.150	0.146
rs4939827	C/T	18	SMAD7	18q21.1	448 (57.7)	279 (36)	49 (6.3)	0.243	0.531
rs5934683	T/C	X	GPR143—SHROOM2	Xp22.2	642 (83.4)	79 (10.3)	49 (6.4)	0.115	0.123
rs6687758	A/G	1	DUSP10—CICP13	1q41	384 (49.7)	321 (41.5)	68 (8.8)	0.296	0.937
rs6983267	T/G	8	SRRM1P1—POU5F1B	8q24.21	205 (27.7)	394 (53.2)	141 (19.1)	0.457	0.047
rs7014346	G/A	8	SRRM1P1—POU5F1B	8q24.21	355 (46.2)	348 (45.3)	65 (8.5)	0.311	0.114
rs7758229	G/T	6	SLC22A3	6q25.3	451 (58.5)	277 (35.9)	43 (5.6)	0.235	0.956
rs9929218	G/A	16	CDH1	16q22.1	570 (73.5)	193 (24.9)	13 (1.7)	0.141	0.468
rs10505477	T/C	8	SRRM1P1—POU5F1B	8q24.21	149 (19.6)	385 (50.7)	226 (29.7)	0.449	0.514
rs11903757	T/C	2	NABP1—SDPR	2q32.3	682 (91.2)	65 (8.7)	1 (0.1)	0.045	0.669
rs2057314	T/C	6	DCBLD1	6q22.1	228 (30)	378 (49.7)	155 (20.4)	0.452	0.942
rs7136702	T/C	12	N/A	12q13	223 (29.3)	365 (47.9)	174 (22.8)	0.468	0.294
rs7315438	C/T	12	TBX3—UBA52P7	12q24.21	260 (34.6)	364 (48.5)	127 (16.9)	0.411	0.983
rs961253	C/A	20	TARDBPP1—BMP2	20p12.3	605 (80)	143 (18.9)	8 (1.1)	0.105	0.889

**Table 3:** Multivariate analysis for survival.

		DFS			OS		
		*P*-value	HR	95% CI	*P*-value	HR	95% CI
**Age, ≥64 years**		**4.6×10** ^**–5**^	**1.77**	**1.35–2.34**	**1.2×10** ^**–8**^	**2.56**	**1.85–3.53**
Male sex		0.336	1.23	0.95–1.61	0.294	1.46	0.81–1.97
**CEA, elevated**		**2.0×10** ^**–4**^	**1.81**	**1.32–2.48**	**0.002**	**1.78**	**1.25–2.55**
**Stage, II/III**		**6.6×10** ^**–13**^	**2.75**	**2.09–3.63**	**7.8×10** ^**–9**^	**2.49**	**1.82–3.39**
Differentiation, well/moderate		0.451	0.71	0.29–1.73	0.569	0.75	0.28–2.03
Primary site, colon		0.142	0.89	0.48–1.21	0.111	0.77	0.51–1.92
***CDKN1A* (rs1321311), TT**	**vs.CC/CT**	**0.050**	**1.90**	**1.00–3.60**	**0.049**	**2.05**	**1.00–4.20**
***RHPN2*(rs10411210), TT**	**vs.GG/GT**	**0.034**	**1.94**	**1.05–3.57**	**0.036**	**2.06**	**1.05–4.05**

## Discussion

The prognostic impact of GWAS-identified genetic variants was investigated in CRC patients who had been surgically treated with curative intent. As a result, the current study demonstrated that the *CDKN1A* rs1321311 and *RHPN2* rs10411210 polymorphisms among the 22 selected variants were significantly associated with survival after adjusting for clinical and pathologic factors.

A GWAS is an investigation of many common genetic variants in different individuals to discover if any variant is associated with a specific trait. GWASs have already been actively applied to explore disease susceptibility, prognosis, and drug response prediction [12]. To date, three recent studies have examined the relationship between GWAS-identified CRC risk variants and the clinical outcomes of CRC [9–11]. In Western countries, Phipps *et al*. examined the relationship between GWAS-identified variants and survival of the 2611 CRC patients, and found a strong association between a SNP (rs4939827) in the SMAD 7 gene and reduced disease-specific survival and OS [10]. Another study based on Western populations evaluated the survival results of 285 stage II or III CRC patients receiving fluorouracil-based chemotherapy, and found an association between one SNP (rs10318) and the recurrence of stage II and between three SNPs (rs10749971, rs961253, and rs355527) and the recurrence of stage III [9]. However, the influence of ethnic variations should also be considered when interpreting the prognostic impact of GWAS-identified variants [13]. Thus, a recent study by Xing *et al*. reported an association between two variants (rs4779584 and rs10795668) and a reduced risk of survival and a marginal effect between rs10795668 and chemotherapy in 380 Chinese CRC patients [11]. Notwithstanding, there are several important differences between the data in the present study and that in previous CRC prognostic studies. First, given the homogeneous ethnic background of Korean patients, any potential confounding effect due to ethnicity is likely to be small in the current study. Moreover, this study included a relatively large number of patients. Furthermore, the adjuvant treatment application was consistent and the loss to follow-up was very low. However, even though the present data identified certain gene variants as a statistically significant prognostic factor for survival in operated CRC, these results should be interpreted cautiously. Considering multiple comparison issue, we cannot rule out the possibility of a type I error in the analysis of individual SNP. Therefore, additional studies with larger sample sizes are required.

For the current study, the TT genotype of *CDKN1A* rs1321311 was significantly associated with worse survival outcomes. *CDKN1A* is a conserved protein belong to the CDK inhibitor Cip/Kip family, that has been implicated in cell cycle regulation [14]. In particular, this molecule is known to be involved in the regulation of fundamental cellular programs, such as cell proliferation, differentiation, migration, senescence, and apoptosis [15]. Although its mechanism and effect on cancer are still unknown, several reports have indicated that *CDKN1A* may be a biologic predictor and beneficial target for cancer treatment using cell cycle alteration [16]. Interestingly, several previous studies have demonstrated an association between a decreased expression of *CDKN1A* and the recurrence of stage II CRC, along with tumor suppression function in the colon of CDKN1A-deficient mice [17,18]. Therefore, the above findings suggest that *CDKN1A* can be a possible biomarker for CRC. Dunlop *et al*. also performed a GWAS meta-analysis to identify common variants influencing CRC risk, including 29778 cases and 29204 controls, and found a strong association between the *CDKN1A* (rs1321311) polymorphism and an increased CRC risk [19]. Similarly, the current study also found an association between the TT genotype and an association with a worse survival. However, there is no previous data on the prognostic role of *CDKN1A* (rs1321311) polymorphisms in CRC. In patients with head and neck cancer, the CT/TT genotype has been associated with a worse second primary malignancy risk than the CC genotype [20].

Another finding from the present study was a significant association between the *RHPN2* rs10411210 TT genotype and poor survival outcomes in patients with surgically resected CRC. *RHPN2* is a master regulator of actin cytoskeleton rearrangement and plays a role in various actin-modulating protein targets [21]. Yet, despite increasing evidence that *RHPN2* plays important roles in several aspects of tumorigenesis and cancer progression, it is still unclear whether the polymorphism itself alters the function of the protein expression [22]. Indeed, several GWASs have already established a significant association between the *RHPN2* polymorphism and an increased risk of CRC [3,23,24]. Thus, the association between CRC risk and the TT genotype is consistent across these studies. However, such previous studies have only focused on the risk of CRC, and not the impact on the survival outcomes of CRC. Consequently, the present results show that the role of the *RHPN2* polymorphism still requires further clarification for predicting the prognosis of CRC.

In conclusion, the current findings indicated that genetic variations of *CDKN1A* and *RHPN2* may influence the prognosis for CRC. However, since the exact mechanism and function of these gene variants have not yet been fully defined, the present findings need to be confirmed in further studies with other populations in order to clarify the association between these polymorphisms and the prognosis of CRC.

## Supporting Information

S1 Table Information of 22 genotypes for overall survival and disease-free survival .
(DOCX)

S2 Table Information of 22 genotypes for overall survival and disease-free survival adjusted to multiple comparison correction .
(DOCX)
