# Supplementary material for: Association between GWAS-Identified Genetic Variations and Disease Prognosis for Patients with Colorectal Cancer
Source: PLoS One. 2015 Mar 23;10(3):e0119649. doi: 10.1371/journal.pone.0119649 (PMC4370892; doi:10.1371/journal.pone.0119649)
Supplement: S1 Table — . (DOCX) [file pone.0119649.s001.docx]

**S1 Table. Information of 22 genotypes for overall survival and disease-free survival**

|  |  | Overall survival | | | | | |  | Disease-free survival | | | | | |  |  |
| --- | --- | --- | --- | --- | --- | --- | --- | --- | --- | --- | --- | --- | --- | --- | --- | --- |
| rsID | type | Dominant | | Recessive | | Codominant | |  | Dominant | | Recessive | | Codominant | |  |  |
|  |  | HR^a^ (95% CI) | *P*-value | HR^a^ (95% CI) | *P*-value | HR^a^ (95% CI) | *P*-value |  | HR^a^ (95% CI) | *P*-value | HR^a^ (95% CI) | *P*-value | HR^a^ (95% CI) | *P*-value |  |  |
| **rs10411210** | **C/T** | **1.29 (0.95-1.75)** | **0.105** | **2.06 (1.05-4.05)** | **0.036** | **1.32 (1.02-1.71)** | **0.038** |  | **1.23 (0.93-1.62)** | **0.141** | **1.94 (1.05-3.57)** | **0.034** | **1.26 (1.00-1.60)** | **0.051** |  |  |
| rs10795668 | G/A | 1.27 (0.94-1.71) | 0.121 | 0.88 (0.55-1.41) | 0.597 | 1.10 (0.89-1.36) | 0.385 |  | 1.18 (0.90-1.53) | 0.229 | 0.90 (0.59-1.37) | 0.629 | 1.06 (0.88-1.29) | 0.519 |  |  |
| rs10936599 | T/C | 1.09 (0.80-1.49) | 0.575 | 1.27 (0.87-1.86) | 0.215 | 1.12 (0.91-1.39) | 0.296 |  | 0.93 (0.71-1.23) | 0.620 | 1.25 (0.89-1.75) | 0.192 | 1.04 (0.85-1.25) | 0.727 |  |  |
| rs11169552 | C/T | 0.82 (0.61-1.11) | 0.206 | 1.15 (0.72-1.84) | 0.562 | 0.92 (0.73-1.16) | 0.492 |  | 0.89 (0.68-1.16) | 0.379 | 1.09 (0.72-1.64) | 0.692 | 0.95 (0.78-1.17) | 0.639 |  |  |
| **rs1321311** | **G/T** | **1.19 (0.86-1.65)** | **0.304** | **2.05 (1.00-4.20)** | **0.049** | **1.24 (0.94-1.63)** | **0.132** |  | **1.19 (0.89-1.59)** | **0.240** | **1.90 (1.00-3.60)** | **0.050** | **1.23 (0.96-1.57)** | **0.101** |  |  |
| rs3802842 | A/C | 0.86 (0.63-1.17) | 0.338 | 0.78 (0.53-1.17) | 0.233 | 0.87 (0.70-1.07) | 0.190 |  | 0.85 (0.64-1.12) | 0.249 | 0.79 (0.56-1.13) | 0.192 | 0.86 (0.72-1.05) | 0.133 |  |  |
| rs3824999 | A/C | 1.39 (0.88-1.91) | 0.062 | 1.05 (0.69-1.60) | 0.807 | 1.19 (0.96-1.47) | 0.114 |  | 1.31 (0.99-1.73) | 0.064 | 1.19 (0.83-1.70) | 0.341 | 1.19 (0.99-1.44) | 0.071 |  |  |
| rs4444235 | C/T | 1.35 (0.97-1.89) | 0.074 | 1.26 (0.92-2.20) | 0.084 | 1.32 (0.91-1.93) | 0.099 |  | 1.26 (0.95-1.68) | 0.116 | 2.40 (0.73-1.90) | 0.135 | 1.56 (1.23-2.48) | 0.127 |  |  |
| rs4779584 | T/C | 1.10 (0.79-1.52) | 0.591 | 1.34 (0.63-2.87) | 0.449 | 1.11 (0.84-1.46) | 0.479 |  | 0.92 (0.68-1.24) | 0.576 | 1.40 (0.72-2.74) | 0.323 | 0.98 (0.76-1.27) | 0.875 |  |  |
| rs4939827 | C/T | 0.90 (0.67-1.22) | 0.496 | 1.14 (0.65-2.01) | 0.647 | 0.96 (0.75-1.22) | 0.716 |  | 0.89 (0.68-1.16) | 0.386 | 1.03 (0.62-1.72) | 0.907 | 0.93 (0.75-1.16) | 0.521 |  |  |
| rs5934683 | T/C | 1.03 (0.69-1.52) | 0.904 | 1.05 (0.55-1.99) | 0.886 | 1.02 (0.77-1.35) | 0.884 |  | 0.99 (0.69-1.42) | 0.957 | 1.04 (0.58-1.86) | 0.907 | 1.00 (0.78-1.29) | 0.991 |  |  |
| rs6687758 | A/G | 0.92 (0.68-1.23) | 0.555 | 1.24 (0.75-2.06) | 0.404 | 0.99 (0.78-1.25) | 0.912 |  | 0.81 (0.62-1.05) | 0.111 | 1.31 (0.84-2.03) | 0.238 | 0.92 (0.75-1.14) | 0.444 |  |  |
| rs6983267 | T/G | 1.37 (0.96-1.95) | 0.083 | 1.09 (0.75-1.59) | 0.640 | 1.17 (0.94-1.45) | 0.157 |  | 1.13 (0.83-1.53) | 0.437 | 1.06 (0.76-1.47) | 0.755 | 1.07 (0.88-1.30) | 0.488 |  |  |
| rs7014346 | G/A | 1.46 (0.81-1.97) | 0.215 | 1.26 (0.77-2.09) | 0.361 | 1.30 (0.64-1.63) | 0.121 |  | 1.23 (0.95-1.61) | 0.124 | 1.23 (0.78-1.93) | 0.375 | 1.18 (0.96-1.45) | 0.110 |  |  |
| rs7758229 | G/T | 1.00 (0.74-1.35) | 0.984 | 0.79 (0.39-1.62) | 0.525 | 0.97 (0.75-1.24) | 0.794 |  | 0.90 (0.68-1.18) | 0.428 | 0.72 (0.38-1.35) | 0.303 | 0.89 (0.71-1.11) | 0.295 |  |  |
| rs9929218 | G/A | 0.80 (0.56-1.13) | 0.205 | 0.00 (0.00-. ) | 0.972 | 0.76 (0.54-1.06) | 0.101 |  | 0.88 (0.65-1.19) | 0.388 | 0.00 (0.00-. ) | 0.968 | 0.82 (0.62-1.09) | 0.177 |  |  |
| rs10505477 | T/C | 0.87 (0.60-1.24) | 0.435 | 0.72 (0.51-1.01) | 0.059 | 0.83 (0.68-1.03) | 0.091 |  | 0.97 (0.70-1.34) | 0.842 | 0.83 (0.62-1.12) | 0.221 | 0.92 (0.76-1.11) | 0.359 |  |  |
| rs11903757 | T/C | 0.80 (0.43-1.47) | 0.467 | 0.00 (0.00-. ) | 0.978 | 0.79 (0.43-1.46) | 0.456 |  | 0.95 (0.57-1.59) | 0.855 | 0.00 (0.00-. ) | 0.974 | 0.95 (0.57-1.58) | 0.830 |  |  |
| rs2057314 | T/C | 0.87 (0.63-1.20) | 0.402 | 0.97 (0.66-1.42) | 0.869 | 0.93 (0.75-1.16) | 0.518 |  | 0.91 (0.68-1.21) | 0.505 | 0.88 (0.63-1.24) | 0.469 | 0.92 (0.76-1.12) | 0.392 |  |  |
| rs7136702 | T/C | 0.85 (0.62-1.17) | 0.311 | 0.93 (0.64-1.34) | 0.690 | 0.91 (0.74-1.12) | 0.378 |  | 1.06 (0.79-1.42) | 0.712 | 0.93 (0.67-1.29) | 0.667 | 1.00 (0.83-1.20) | 0.991 |  |  |
| rs7315438 | C/T | 0.80 (0.58-1.09) | 0.151 | 1.03 (0.69-1.52) | 0.903 | 0.90 (0.73-1.13) | 0.364 |  | 0.81 (0.61-1.06) | 0.129 | 0.93 (0.65-1.33) | 0.685 | 0.88 (0.73-1.07) | 0.211 |  |  |
| rs961253 | C/A | 1.09 (0.75-1.58) | 0.672 | 1.45 (0.36-5.89) | 0.601 | 1.10 (0.77-1.55) | 0.610 |  | 1.15 (0.83-1.59) | 0.416 | 0.97 (0.24-3.91) | 0.965 | 1.12 (0.83-1.52) | 0.457 |  |  |
| ^a^Hazard ratio were calculated using multivariate Cox proportional hazard models, adjusted for age, grade of differentiation, sex, primary site, pathologic stage, preoperative CEA level. | | | | | | | | | | | | | | | | |
