# Supplementary material for: Association between GWAS-Identified Genetic Variations and Disease Prognosis for Patients with Colorectal Cancer
Source: PLoS One. 2015 Mar 23;10(3):e0119649. doi: 10.1371/journal.pone.0119649 (PMC4370892; doi:10.1371/journal.pone.0119649)
Supplement: S2 Table — . (DOCX) [file pone.0119649.s002.docx]

**S2 Table. Information of 22 genotypes for overall survival and disease-free survival adjusted to multiple comparison correction**

|  |  | Overall survival | | |  | | Disease-free survival | | |
| --- | --- | --- | --- | --- | --- | --- | --- | --- | --- |
| rsID |  | Corrected  *P*-value | | |  | Corrected  *P*-value | | | |
|  |  | Bonferroni’ method | Hochberg’s method | False discovery rate |  | Bonferroni’ method | | Hochberg’s method | False discovery rate |
| **rs10411210** | **Dominant** | **1** | **0.998** | **0.594** |  | **1** | | **0.998** | **0.594** |
|  | **Recessive** | **1** | **0.998** | **0.594** |  | **1** | | **0.998** | **0.594** |
|  | **Codominant** | **1** | **0.998** | **0.594** |  | **1** | | **0.998** | **0.594** |
| rs10795668 | Dominant | 1 | 0.998 | 0.594 |  | 1 | | 0.998 | 0.61013 |
|  | Recessive | 1 | 0.998 | 0.82326 |  | 1 | | 0.998 | 0.82264 |
|  | Codominant | 1 | 0.998 | 0.61013 |  | 1 | | 0.998 | 0.68588 |
| rs10936599 | Dominant | 1 | 0.998 | 0.82264 |  | 1 | | 0.998 | 0.64653 |
|  | Recessive | 1 | 0.998 | 0.88943 |  | 1 | | 0.998 | 0.82264 |
|  | Codominant | 1 | 0.998 | 0.88568 |  | 1 | | 0.998 | 0.82264 |
| rs11169552 | Dominant | 1 | 0.998 | 0.594 |  | 1 | | 0.998 | 0.726 |
|  | Recessive | 1 | 0.998 | 0.82264 |  | 1 | | 0.998 | 0.82264 |
|  | Codominant | 1 | 0.998 | 0.75342 |  | 1 | | 0.998 | 0.82264 |
| **rs1321311** | **Dominant** | **1** | **0.998** | **0.81876** |  | **1** | | **0.998** | **0.7458** |
|  | **Recessive** | **1** | **0.998** | **0.594** |  | **1** | | **0.998** | **0.594** |
|  | **Codominant** | **1** | **0.998** | **0.61013** |  | **1** | | **0.998** | **0.594** |
| rs3802842 | Dominant | 1 | 0.998 | 0.82264 |  | 1 | | 0.998 | 0.82264 |
|  | Recessive | 1 | 0.998 | 0.85412 |  | 1 | | 0.998 | 0.82264 |
|  | Codominant | 1 | 0.998 | 0.82264 |  | 1 | | 0.998 | 0.82264 |
| rs3824999 | Dominant | 1 | 0.998 | 0.61013 |  | 1 | | 0.998 | 0.61013 |
|  | Recessive | 1 | 0.998 | 0.82264 |  | 1 | | 0.998 | 0.87818 |
|  | Codominant | 1 | 0.998 | 0.81876 |  | 1 | | 0.998 | 0.68588 |
| rs4444235 | Dominant | 1 | 0.998 | 0.61013 |  | 1 | | 0.998 | 0.81876 |
|  | Recessive | 1 | 0.998 | 0.594 |  | 1 | | 0.998 | 0.61013 |
|  | Codominant | 1 | 0.998 | 0.594 |  | 1 | | 0.998 | 0.61013 |
| rs4779584 | Dominant | 1 | 0.998 | 0.82264 |  | 1 | | 0.998 | 0.82326 |
|  | Recessive | 1 | 0.998 | 0.61013 |  | 1 | | 0.998 | 0.594 |
|  | Codominant | 1 | 0.998 | 0.75342 |  | 1 | | 0.998 | 0.88568 |
| rs4939827 | Dominant | 1 | 0.998 | 0.76048 |  | 1 | | 0.998 | 0.76048 |
|  | Recessive | 1 | 0.998 | 0.85017 |  | 1 | | 0.998 | 0.84954 |
|  | Codominant | 1 | 0.998 | 0.82264 |  | 1 | | 0.998 | 0.82264 |
| rs5934683 | Dominant | 1 | 0.998 | 0.75342 |  | 1 | | 0.998 | 0.82264 |
|  | Recessive | 1 | 0.998 | 0.95265 |  | 1 | | 0.998 | 0.88943 |
|  | Codominant | 1 | 0.998 | 0.82264 |  | 1 | | 0.998 | 0.88568 |
| rs6687758 | Dominant | 1 | 0.998 | 0.82264 |  | 1 | | 0.998 | 0.594 |
|  | Recessive | 1 | 0.998 | 0.82264 |  | 1 | | 0.998 | 0.76676 |
|  | Codominant | 1 | 0.998 | 0.87818 |  | 1 | | 0.998 | 0.7458 |
| rs6983267 | Dominant | 1 | 0.998 | 0.594 |  | 1 | | 0.998 | 0.81876 |
|  | Recessive | 1 | 0.998 | 0.73721 |  | 1 | | 0.998 | 0.82264 |
|  | Codominant | 1 | 0.998 | 0.594 |  | 1 | | 0.998 | 0.75342 |
| rs7014346 | Dominant | 1 | 0.998 | 0.594 |  | 1 | | 0.998 | 0.7416 |
|  | Recessive | 1 | 0.998 | 0.61013 |  | 1 | | 0.998 | 0.64653 |
|  | Codominant | 1 | 0.998 | 0.594 |  | 1 | | 0.998 | 0.61013 |
| rs7758229 | Dominant | 1 | 0.998 | 0.84954 |  | 1 | | 0.998 | 0.7458 |
|  | Recessive | 1 | 0.998 | 0.82264 |  | 1 | | 0.998 | 0.7458 |
|  | Codominant | 1 | 0.998 | 0.82264 |  | 1 | | 0.998 | 0.64653 |
| rs9929218 | Dominant | 1 | 0.998 | 0.61013 |  | 1 | | 0.998 | 0.81876 |
|  | Recessive | 1 | 0.998 | 0.594 |  | 1 | | 0.998 | 0.594 |
|  | Codominant | 1 | 0.998 | 0.594 |  | 1 | | 0.998 | 0.61013 |
| rs10505477 | Dominant | 1 | 0.998 | 0.61013 |  | 1 | | 0.998 | 0.81876 |
|  | Recessive | 1 | 0.998 | 0.594 |  | 1 | | 0.998 | 0.61013 |
|  | Codominant | 1 | 0.998 | 0.594 |  | 1 | | 0.998 | 0.61013 |
| rs11903757 | Dominant | 1 | 0.998 | 0.594 |  | 1 | | 0.998 | 0.6313 |
|  | Recessive | 1 | 0.998 | 0.82264 |  | 1 | | 0.998 | 0.82264 |
|  | Codominant | 1 | 0.998 | 0.594 |  | 1 | | 0.998 | 0.61013 |
| rs2057314 | Dominant | 1 | 0.998 | 0.82264 |  | 1 | | 0.998 | 0.90425 |
|  | Recessive | 1 | 0.998 | 0.998 |  | 1 | | 0.998 | 0.82264 |
|  | Codominant | 1 | 0.998 | 0.83217 |  | 1 | | 0.998 | 0.82264 |
| rs7136702 | Dominant | 1 | 0.998 | 0.61013 |  | 1 | | 0.998 | 0.998 |
|  | Recessive | 1 | 0.998 | 0.75342 |  | 1 | | 0.998 | 0.82264 |
|  | Codominant | 1 | 0.998 | 0.61013 |  | 1 | | 0.998 | 0.82264 |
| rs7315438 | Dominant | 1 | 0.998 | 0.7458 |  | 1 | | 0.998 | 0.7416 |
|  | Recessive | 1 | 0.998 | 0.82264 |  | 1 | | 0.998 | 0.998 |
|  | Codominant | 1 | 0.998 | 0.82264 |  | 1 | | 0.998 | 0.82264 |
| rs961253 | Dominant | 1 | 0.998 | 0.82264 |  | 1 | | 0.998 | 0.82264 |
|  | Recessive | 1 | 0.998 | 0.82264 |  | 1 | | 0.998 | 0.95265 |
|  | Codominant | 1 | 0.998 | 0.82264 |  | 1 | | 0.998 | 0.82264 |
